# Nestin(+) Tissue-Resident Multipotent Stem Cells Contribute to Tumor Progression by Differentiating into Pericytes and Smooth Muscle Cells Resulting in Blood Vessel Remodeling

**DOI:** 10.3389/fonc.2014.00169

**Published:** 2014-06-26

**Authors:** Diana Klein, Nicole Meissner, Veronika Kleff, Holger Jastrow, Masahiro Yamaguchi, Süleyman Ergün, Verena Jendrossek

**Affiliations:** ^1^Institute for Cell Biology (Cancer Research), University Hospital Essen, University of Duisburg-Essen, Essen, Germany; ^2^Institute of Anatomy, University Hospital, University of Duisburg-Essen, Essen, Germany; ^3^Department of Physiology, Graduate School of Medicine, University of Tokyo, Tokyo, Japan; ^4^Institute of Anatomy and Cell Biology, University of Würzburg, Würzburg, Germany

**Keywords:** nestin, mesenchymal stem cell, pericytes, vascular remodeling, vessel maturation

## Abstract

Tumor vessels with resistance to anti-angiogenic therapy are characterized by the normalization of the vascular structures through integration of mature pericytes and smooth muscle cells (SMC) into the vessel wall, a process termed vessel stabilization. Unfortunately, stabilization-associated vascular remodeling can result in reduced sensitivity to subsequent anti-angiogenic therapy. We show here that blockade of VEGF by bevacizumab induces stabilization of angiogenic tumor blood vessels in human tumor specimen by recruiting Nestin-positive cells, whereas mature vessels down-regulated Nestin-expression. Using xenograft tumors growing on bone-marrow (BM) chimera of C57Bl/6 wildtype and Nestin-GFP transgenic mice, we show for first time that Nestin(+) cells inducing the maturation of tumor vessels do not originate from the BM but presumably reside within the adventitia of adult blood vessels. Complementary *ex vivo* experiments using explants of murine aortas revealed that Nestin(+) multipotent stem cells (MPSCs) are mobilized from their niche and differentiated into pericytes and SMC through the influence of tumor-cell-secreted factors. We conclude that tissue-resident Nestin(+) cells are more relevant than BM-derived cells for vessel stabilization and therefore have to be considered in future strategies for anti-angiogenic therapy. The identification of proteins mediating recruitment or differentiation of local Nestin(+) cells with potential stem cell character to angiogenic blood vessels may allow the definition of new therapeutic targets to reduce tumor resistance against anti-angiogenic drugs.

## Introduction

Abnormal vasculature is a hallmark of solid tumors. Deregulated formation of new blood vessels leads to a pre-dominance of immature blood vessels with patchy endothelial lining. Moreover, the newly formed vessels cannot actively respond to physiological stimuli because they lack the smooth muscle elements in their walls (1). Consequently, tumor vessels prove to be functionally inferior. The resulting undirected blood flow with areas of oversupply as well as areas of undersupply complicates an efficient administration of intravenous drugs in cancer therapy (2). However, the vascular network can mature by recruitment of pericytes and smooth muscle cells (SMC) to stabilize the immature tumor vessels and this process is accelerated when anti-angiogenic agents were used (3, 4). Though there is some evidence that pericytes and SMC might originate from multipotent mesenchymal stem cells the origin of these mural cells and the molecular changes associated with pericyte-mediated vascular remodeling are still poorly understood (5, 6).

Angiogenesis inhibitors such bevacizumab are used as anti-cancer agents and decrease tumor growth. Bevacizumab is a humanized monoclonal anti-VEGF antibody that prevents the interaction of VEGF with the corresponding VEGF receptors by neutralizing all VEGF isoforms (7, 8). Paradoxically, anti-angiogenic therapies promote vascular remodeling within the respective tumors resulting in stabilization of the newly formed tumor blood vessels (9, 10). Herein, normalization is achieved by the recruitment and integration of mature pericytes into the vessel wall for capillaries as well as SMC for larger vessels (11, 12). Notably, remodeling of the tumor vasculature leads to increased resistance of stabilized vessels to further anti-angiogenic therapies (10, 13).

Consequently, there is an urgent need for the development of second-generation anti-angiogenic therapies. Such therapies may balance initial vascular stabilization with a subsequent de-stabilization to make the remaining vessels susceptible to a second wave of anti-angiogenic therapy (14). Thus, targeting both, endothelial cells and pericytes, could be an alternative promising approach to improve anti-angiogenic treatment for malignant tumors (15).

An improved understanding of the origin of pericytes and SMC and of the signals driving their recruitment and differentiation is necessary to efficiently target stabilization-associated vascular remodeling to improve anti-angiogenic anti-cancer therapies. We recently demonstrated that vascular wall-resident CD44(+) multipotent stem cells (VW-MPSCs) within the adult human vascular adventitia are capable to differentiate into pericytes and SMC (5). We assume that on the basis of their anatomic location such vessel-resident stem cells are available as first point of contact for tumor cells and secreted factors (16, 17).

Here, we aimed to gain insight into the use of Nestin as a marker for the origin of mural pericytes and SMCs in vascular stabilization processes during tumor progression. For this, we first examined Nestin-expression in human tumor specimen. Moreover, we used the transgenic Nestin-GFP mice as well as bone-marrow (BM) chimera of C57Bl/6 wild type (wt) and Nest-GFP mice to trace the recruitment Nestin(+) MPSCs during vascular remodeling of tumor blood vessels in xenograft tumors and to analyze the relative importance of tissue-resident Nestin(+) MPSCs vs. circulating (BM-derived) MPSC. Our data demonstrate that tissue-resident Nestin(+) progenitor cells are indeed the major source for pericytes and SMC in vascular remodeling processes. The identification of Nestin as a novel marker for mural pericytes will facilitate future investigations about the mechanisms driving their recruitment and differentiation.

## Materials and Methods

### Reagents and antibodies

Nestin antibody was from Acris Antibodies (Herford, Germany), alpha smooth muscle actin (SMA) and CD34 antibodies were from Santa Cruz (Santa Cruz, USA), CD31 antibody was from Dianova (Hamburg, Germany), and GFP antibody was from Thermo Scientific (Dreieich, Germany). Peroxidase and fluorescence-labeled secondary antibodies were from Jackson IR Laboratories (West Grove, USA).

### Human tumor tissue

Tissues from human liver metastases were taken at clinical relapse in patients having developed colorectal adenocarcinoma and undergoing neoadjuvant treatment using bevacizumab and regular chemotherapy and were obtained during surgery according to local ethical and biohazard regulations. Surgical pathology diagnoses were made based on current WHO criteria. Resected tissue specimens were processed for further pathological diagnostic routine in a standardized way as previously described (14). The local ethical review committee of the University Hospital Essen approved all studies including human tissue specimen. Informed consent (written form, Nr. 10-4363) was obtained.

### Cells and tumor mouse model

The mouse melanoma cell line B16F10 and the Lewis lung carcinoma (LLC) cell line was obtained from ATTC (Rockville, MD, USA). Cells were cultured in DMEM with 10% FCS, 100 U/mL penicillin and streptomycin in a humidified atmosphere (5% CO2 at 37°C) and passaged twice a week. A mouse tumor model was used as described before (18). In brief, BM cells were harvested aseptically by flushing the tibias and femurs of adult animals and subjected to erythrocytes lysis. C57Bl/6J mice were lethally irradiated with a split dose (7 + 3 Gy) of a X-ray source and were intravenously transplanted with 1 × 10^6^ unfractionated murine Nest-GFP-expressing BM cells from Nest-GFP transgenic donor mice into the tail vain (19). B16F10 and LLC cells were harvested by a brief trypsinization (0.05% trypsin/0.02% EDTA), washed, and resuspended in PBS at a density of 1 × 10^7^ cells/mL. After hematopoietic reconstitution, B16F10 or LLC cells were subcutaneously implanted into the flank of the mice. Up to 20 animals of each experimental group received a single subcutaneous injection of 1 × 10^6^ viable tumor cells. After around 21 days of tumor growth animals were sacrificed and tumors were harvested and subdivided in four parts. One part was freshly frozen in OCT Tissue Tek (Sakura) for immunofluorescence staining, the second part was fixed in 4% PFA and paraffin embedded for immunohistochemical staining, the other two parts were fresh frozen in liquid nitrogen and prepared for protein or RNA analyses. In addition, a vice-versa experiment was performed. Here, Nest-GFP mice were lethally irradiated and BM cells from C57Bl/6 wt donor mice were transplanted. Experiments were repeated three times. All procedures involving mice were conducted in accordance with protocols approved by the local institutional Animal Care Committee (Regierungspräsidium Düsseldorf Az 84-02.04.2012.A137; 84-02.04.2012.A034).

### Hematopoietic engraftment analysis

To confirm the hematopoietic engraftment, peripheral blood analyses were performed via blood count measurement (VetABC) and flow cytometry. Blood samples were obtained from tail vein before tumor implantation and from heart when the mice were sacrificed, and subjected to erythrocyte lyses. Engraftment was accessed by the percentage of GFP(+) cells in blood samples and in BM. Samples were examined in two-color FACS using Cellquest software (Becton Dickinson, Heidelberg, Germany).

### Isolation and purification of tissue-resident-MPSCs

Mice were scarified after transcardial perfusion with PBS and aortas were removed. Isolated aortas were excised under a dissection microscope and contaminating fatty and muscle tissue was removed. After several washes, vessels were mechanically minced and dissociated for 10–15 min at 37°C in OptiMEM I medium (GIBCO) containing 0.2% type 2-collagenase (Worthington, Lakewood, USA). On dissociation, cells were washed twice in PBS containing 5% FCS (300 × *g*, 10 min, 4°C). Cellular suspensions were passed through 70 μm pore size filters. Nest-GFP-expressing cells were further purified using fluorescent activated cell sorting based on their GFP-expression and plated in 96 well plastic dishes (one cell per well). Primary Nest-GFP cells were cultivated using complete DMEM supplemented with 20% FCS.

### Trilineage differentiation assay

Differentiation of cultivated MPSCs into adipocytes, chondrocytes, and osteocytes was done using ready-to-use differentiation media from Lonza (hMSC Differentiation BulletKit – Adipogenic, PT-3004; – Chondrogenic, PT-3003; – Osteogenic, PT-3002) according to the manufactures instructions. Adipogenic differentiation was verified using Oil red staining, chondrogenic differentiation was verified using Collagen type II antibody (Santa Cruz) and immunohistochemitry and osteogenic differentiation was verified using NBT/BCIP staining (Sigma) for alkaline phosphatase activity.

### Conditioned media

B16F10 and LLC cells were cultured as described above. When cells were grown to confluence, media were replaced and cells were cultured in the presence of 0.5% fetal bovine serum for 24 h before collection of media. Control medium was incubated for the same time in the incubator without cells.

### Arterial ring assay

Isolated mouse aortas were cut into 2 mm thick rings and placed into a 48-well tissue culture plate within PureCol^®^ collagen-gel (Advanced BioMatrix, Denver). After polymerization normal growth medium (EGM-2 medium, PromoCell) was added and the rings were further incubated at 37°C in normal growth medium alone, or supplemented with conditioned medium (ratio 1:1). The medium was changed every third day. Capillary outgrowth was analyzed using light microscopy at indicated time points.

### Immunohistochemistry and immunofluorescence

Paraffin embedded tissue sections were hydrated using a descending alcohol series, incubated for 10–20 min in target retrieval solution (Dako) and incubated with blocking solution (2% FCS/PBS). After permeabilization, sections were incubated with primary antibodies over night at 4°C. Antigen was detected with a peroxidase-conjugated secondary antibody (1/250) and DAB staining (Dako). Nuclei were counterstained using hematoxylin. For immunofluorescence analysis, the antigen was detected with an anti-rabbit-Alexa488 and anti-mouse-Alexa555-conjugated secondary antibody (1/500). Hoechst 33342 (Invitrogen, Karlsruhe, Germany) was used for nuclei staining. Specimens were analyzed by confocal microscopy. Quantification was done by counting numbers of specific immunoreactive structures in four randomly chosen optical fields using the indicated microscopes. Data are presented as mean ± SEM from four independent experiments.

### Western blot

Whole cell lysates were generated by scraping cells into ice-cold RIPA-P buffer (150 mmol/L NaCl, 1% NP40, 0.5% sodium-desoxycholate, 0.1% sodium-dodecylsulfate, 50 mmol/L Tris/HCL pH 8, 10 mmol/L NaF, 1 mmol/L Na_3_VO_4_), supplemented with complete Protease-Inhibitor-Cocktail (Roche) and performing 2–3 freeze-thaw cycles. Protein samples (50–100 μg total protein) were subjected to SDS-PAGE electrophoresis and Western blots were done as previously described using GFP (1/1000) or beta-actin (1/5000) antibodies (20).

### Electron microscopy

Tumor tissues were fixed with cacodylat buffered glutaraldehyde (2.5%), contrasted with 1% osmium tetroxide and 1% uranylacetate and embedded in EPON. Thin sections (70 nm) were cut and mounted on 200 Mesh hexagonal cooper grids. For contrast enhancement uranylacetate and lead citrate were applied. Sections were analyzed using a Zeiss transmission electron microscope (EM 902A) at 8 KV. Digital image acquisition was performed on a Morula slow-scan-CCD camera connected to a PC running ITEM 5.0 software.

### Statistical analysis

Paired or unpaired two-tailed *t*-tests were performed using GraphPad InStat3 software depending on effective matching of analyzed data. SD or SEM are indicated by error bars. Significance was assumed for *p*-values ≤ 0.05, * ≤0.05, and ** ≤0.01.

## Results

### Nestin-expression serves as novel selective marker for vascular mural cells

**Figure 1:**
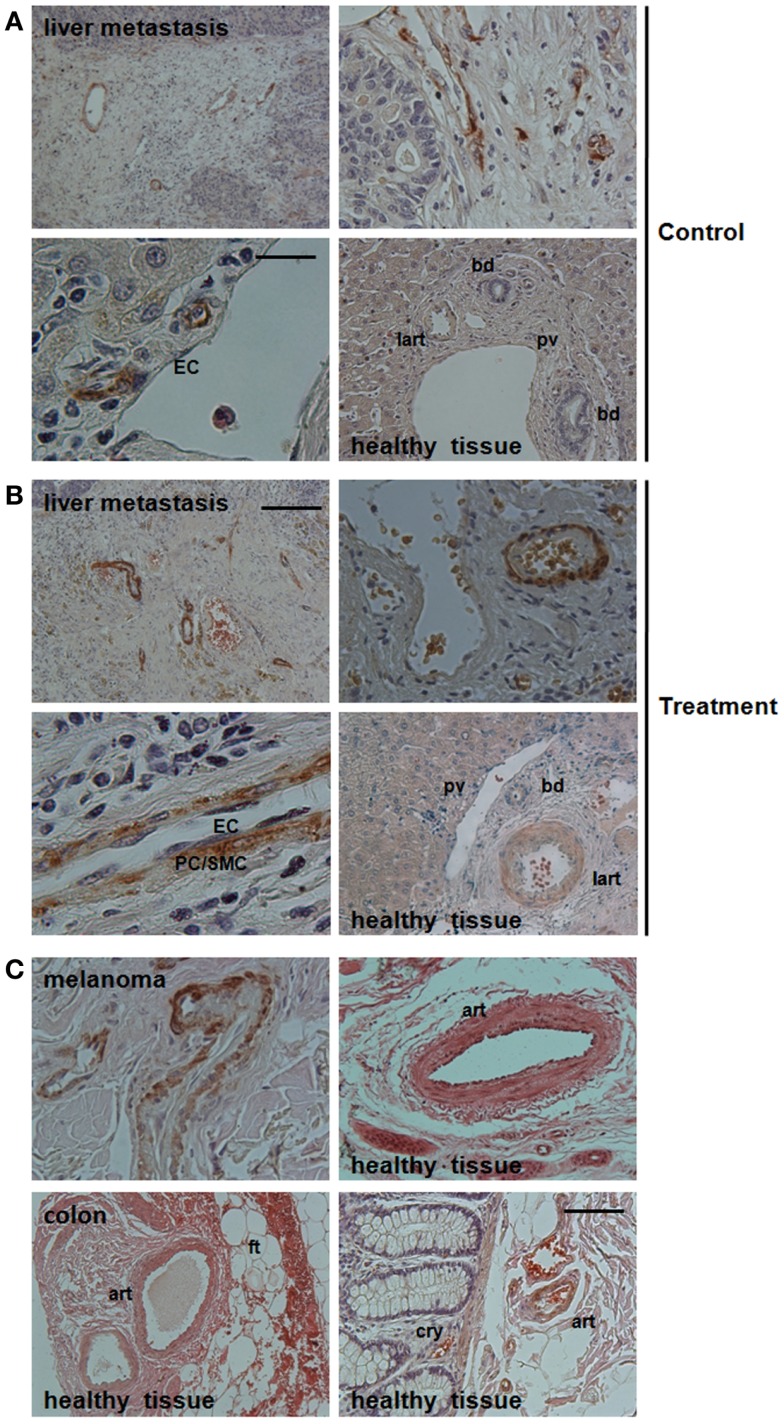
**Immunohistological analysis of Nestin-expression in vessel structures in bevacizumab-treated human tumor tissues**. **(A)** Paraffin-sections of liver metastases from colon carcinoma patients without neoadjuvant treatment (Control) were stained for Nestin. Scale bar 50 μm. **(B)** Treatment group: sections of liver metastases from colon carcinoma patient having received bevacizumab. Representative images of 19 tissue samples are shown (9 treatment and 10 control samples). Scale bar 20 μm. **(C)** Paraffin-sections of human melanoma tissue sections as well as human colon tissue sections were stained for Nestin. Sections were counterstained with hematoxylin. Art, artery; lart, liver artery; pv, portal vein; bd, bile duct; ft, fatty tissue; cry, crypt. Scale bar 10 μm.

In order to investigate, if Nestin-expression might serve as novel and selective marker for newly formed tumor blood vessels, we analyzed angiogenic vessels in tumor specimen from cancer patients (Figure 1). Human liver metastases were collected at clinical relapse in colorectal cancer patients with or without anti-angiogenic treatment with bevacizumab. Interestingly, Nestin(+) cells were mobilized to the newly formed blood vessels suggesting that these cells might have been derived from Nestin(+) tissue-resident MPSCs (Figure 1A). In bevacizumab-treated tumor specimen a more intense expression of Nestin in vascular mural cells and a more regular arrangement of Nestin(+) cells around newly formed tumor blood vessels was detected when compared to untreated tumor tissue, indicating vessel stabilization, maturation, and thus normalization of the vascular bed upon anti-angiogenic treatment (Figure 1B). In contrast, mature vessels were either negative or only slightly positive for Nestin. This suggests, that upon differentiation into SMC Nestin(+) tissue-resident MPSCs down-regulate Nestin-expression.

A detailed morphological analysis showed that Nestin-expression was restricted to the mural cells, particularly pericytes (Figures 1A,B). Of note, Nestin could not be detected in endothelial cells of newly formed tumor blood vessels. This result was confirmed by the lack of co-localization of CD31 and Nestin immunoreactivity in immunofluorescence analysis (not shown).

Similar expression patterns were observed in vessels of human melanoma samples: angiogenic vessels within the tumor were stabilized by Nestin(+) cells, whereas mature vessels in tumor-surrounding tissue down-regulated Nestin-expression (Figure 1C).

### Tissue-resident GFP(+) pericytes closely associate with angiogenic vessels in tumors grown in Nest-GFP transgenic mice

**Figure 2:**
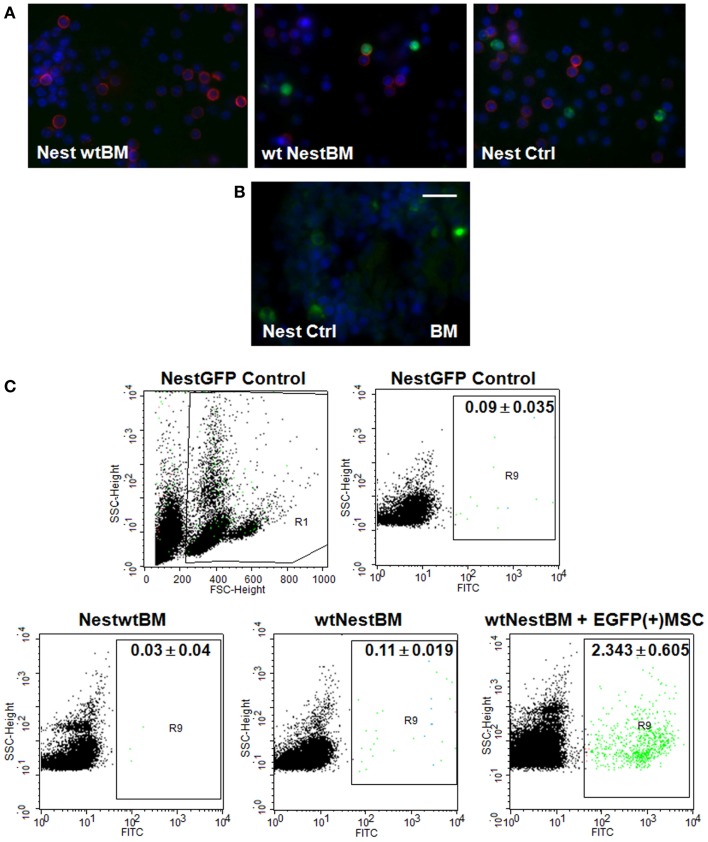
**Nest-GFP+ cells in the peripheral blood and BM of Nest-GFP mice**. **(A)** Nest-GFP transgenic mice were lethally irradiated with a split dose of 7 + 3 Gy total body irradiation and subsequently adoptively transferred with 2 × 10^6^ murine wt BM cells from C57BL/6 donor mice into the tail vain (NestwtBM) and vice-versa (wtNestBM). One group of irradiated animals received NestBM for hematopoietic reconstitution which was supplemented with cultured EGFP(+) MSCs derived from the bone-marrow of C57BL/6-Tg(CAG-EGFP)1Osb/J mice [NestBM + EGFP(+) MSC]. After hematopoietic reconstitution (6–8 weeks after transplantation) blood samples were taken, erythrocytes were removed, and analyzed for GFP-expression by immunofluorescence. Lymphocytes are stained in red. **(B)** Paraffin-sections of Nest-GFP BM specimen were stained for GFP-expression. **(C)** The blood analysis showed a complete engraftment of donor BM by the donor BM-derived Nest-GFP transplanted progenitor cells and vice-versa as indicated by GFP-expression. Control, normal Nest-GFP transgenic mice.

Next, we analyzed the putative contribution of tissue-resident MPSCs as compared to BM-derived MSCs to the formation of newly formed tumor blood vessels. We performed a BM transplantation (BMT) experiment. To track tissue-derived cells *in vivo*, wt BM cells were isolated from C57BL/6 mice and transplanted into lethally irradiated age-matched, syngenic, Nest-GFP transgenic recipients (NestwtBM), and vice-versa. Peripheral blood analysis was done 6 weeks post-transplantation and showed a complete engraftment of implanted donor BM as shown by the reconstitution of BM-GFP(+) cells of wt transplanted littermates with GFP(+) cells (Figure 2, wtNestBM). This indicates a stable replacement of original host stem cell population by donor cells.

**Figure 3:**
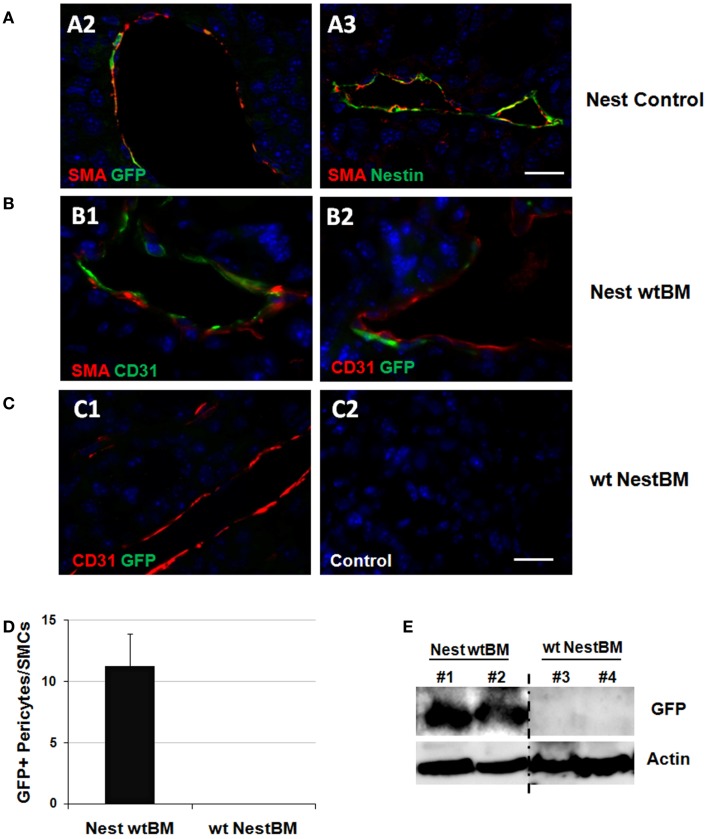
**Tissue-resident Nest-GFP(+) cells and not BM-derived MSCs contribute to remodeling of tumor blood vessels**. **(A)** B16F10 tumor cells were subcutaneously transplanted into the flank of Nest-GFP mice. Tumors were removed 21 days after tumor induction and subjected to immunofluorescence analysis. Vessels were stained for CD31 (endothelial cells), smooth muscle actin (SMA; pericytes/SMC), and GFP or Nestin, respectively. Control sections were stained with isotype controls. Nest-Control, Nest-GFP transgenic mice. Scale bar 15 μm. **(B)** Immunofluorescence analysis of B16F10 tumors grown subcutaneously in the flank of BM-reconstituted Nest-GFP mice. NestwtBM, Nest-GFP mice received bone-marrow (BM) from wt (C57Bl/6) mice after lethally total body irradiation (7 + 3Gy). **(C)** Experiment with reciprocal BM chimera as shown in **(B)**. wtNestBM, wt mice received bone-marrow (BM) from Nest-GFP mice after lethally total body irradiation. Scale bar 20 μm. **(D)** GFP-expression was quantified by counting GFP- and SMA-immunoreactive vascular structures (pericytes/SMC-stabilized vessels). **(E)** GFP-expression was further analyzed using Western blot analysis.

**Figure 4:**
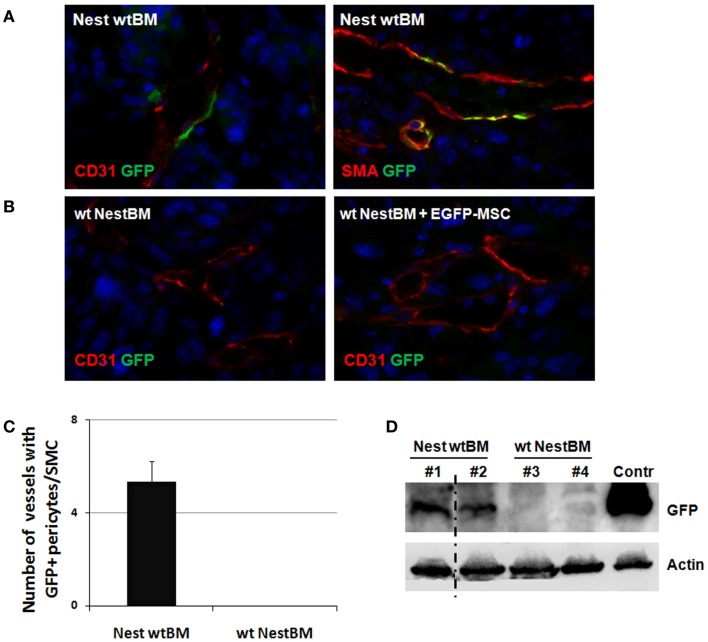
**Tissue-resident Nest-GFP(+) pericytes associate with angiogenic vessels in LLC tumors grown in Nestin-GFP mice**. **(A)** Immunofluorescence analysis of LLC tumors grown subcutaneously in the flank of wt BM-reconstituted Nest-GFP mice (NestwtBM). Tumors were isolated at day 21 and subjected for immunofluorescence analysis. Vessels were stained for CD31 or SMA and GFP. **(B)** Experiment with reciprocal BM chimera as shown in **(A)**. wtNestBM, wt mice having received BM from Nest-GFP mice after total body irradiation. To further confirm that tissue-resident MSCs and not BM-derived MSCs contribute to vascular remodeling processes one group of irradiated animals received NestBM for hematopoietic reconstitution that was supplemented with BM-derived EGFP-labeled MSC. Control sections were stained with isotype controls. Scale bar 20 μm. **(C,D)** GFP-expression was quantified by counting GFP and SMA-immunoreactive vessels and further analyzed using Western blot analysis.

Subcutaneously grown B16F10 melanomas and LLC were then generated by implantation of these cells into the flank of reconstituted mice as well as in untreated Nest-GFP control mice (Figure 3, B16F10 and Figure 4, LLC).

Tumor and surrounding tissues were removed at day 21 and subjected to immunofluorescence analysis. Interestingly, many Nest-GFP(+) cells were detected in the tumor bed of B16F10 tumors and were closely associated with newly formed CD31(+) blood vessels. These cells resembled differentiated SMA(+) pericytes indicative for blood vessel maturation (Figure 3A). Importantly, in the reconstituted animals GFP(+) cells lining the tumor vessels were only detected in NestwtBM mice but not in wtNestBM mice indicating that tissue-resident and not BM-derived cells serve as source for pericytes in vascular remodeling processes (Figures 3B,C). Notably, no co-localization of GFP(+) cells with endothelial markers could be found.

Quantification of Nest-GFP(+) cells associated with newly formed blood vessels in NestwtBM mice and wtNestBM mice indicated that all GFP(+) cells were derived from the host (Figure 3D). In tumor adjacent tissue, only single GFP(+) cells were detected close to larger vessels, in association to myofibrils or in tissue fat (not shown). Of note, the vast majority of SMA(+) pericytes and SMC expressed GFP(+) indicating their origin from the host. In line with these findings, the GFP protein was only detected in Westernblot analyses of tumors grown in Nest-GFP animals reconstituted with BM from wt mice (NestwtBM) but not in wt mice reconstituted with BM of Nestin transgenic mice (wtBMNest). These findings corroborate that tissue-resident and not BM-derived GFP(+) pericytes stabilize angiogenic tumor vessels (Figure 3E).

To corroborate our findings in another tumor model, analogous analyses were performed with the LLC xenograft tumors. As shown in Figure 4, a similar pattern of Nest-GFP-expression was observed in LLC tumors grown on BM chimera of C57Bl/6 wt and Nest-GFP transgenic mice: many Nest-GFP(+) cells were tightly associated with newly formed CD31(+) vascular structures (Figure 4A). Moreover, GFP(+) cells surrounding tumor vessels were only detected in NestwtBM.

To further confirm that tissue-resident GFP(+) cells and not BM-derived cells contribute to vascular remodeling processes one group of irradiated wt animals received NestBM for hematopoietic reconstitution, which was supplemented with BM-derived EGFP-labeled MSC (Figure 4B). Again, no GFP(+) cells could be detected in tumor specimen.

**Figure 5:**
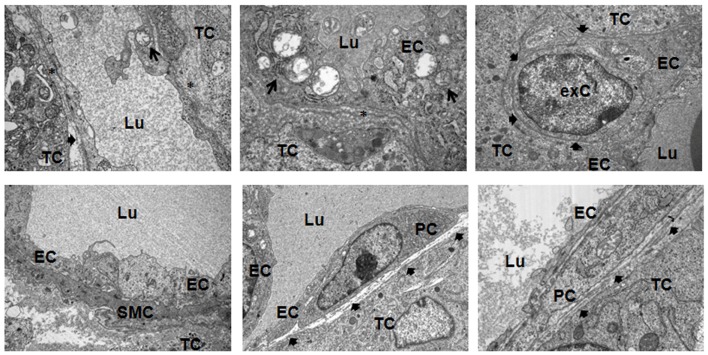
**Ultrastructural analysis of angiogenic tumor vessels**. Subcutaneously grown B16F10 tumors were removed 21 days after tumor induction and subjected to electron microscopic analysis. A defective and irregular basement membrane (bMem) lining angiogenic endothelial cells (EC) is emphasized by an asterix (*), a regular bMem assembly is depicted by a bold arrowhead. Arrows highlight partially degraded mitochondria in EC. PC, pericyte; SMC, smooth muscle cell; TC, tumor-cell; exC, extravasated cell; Lu, lumen. Representative images of three independent experiments are shown.

Subsequent electron microscopic analysis further indicated that pericytes and SMCs not only associate to but properly integrate into tumor blood vessels (Figure 5). At the structural level, the pericytes assembled to new capillaries. Moreover, in some tumor vessels they seemed to be more regularly integrated into the wall of new capillaries because of their tight contact to the endothelial cells, e.g., sharing the same basement membrane (bMem), indicating vessel stabilization and maturation (Figure 7, bold arrows).

Taken together, the vast majority of SMA(+) pericytes expressed GFP(+) in reconstituted NestwtBM mice. This indicated that tissue-resident Nest-GFP(+) pericytes stabilize angiogenic vessels in tumors grown in Nest-GFP mice and highlight the general relevance of these cells in tumor neovascularization.

### Nest-GFP(+) cells predominantly reside in close association with arteries and exhibit typical MSC characteristics

**Figure 6:**
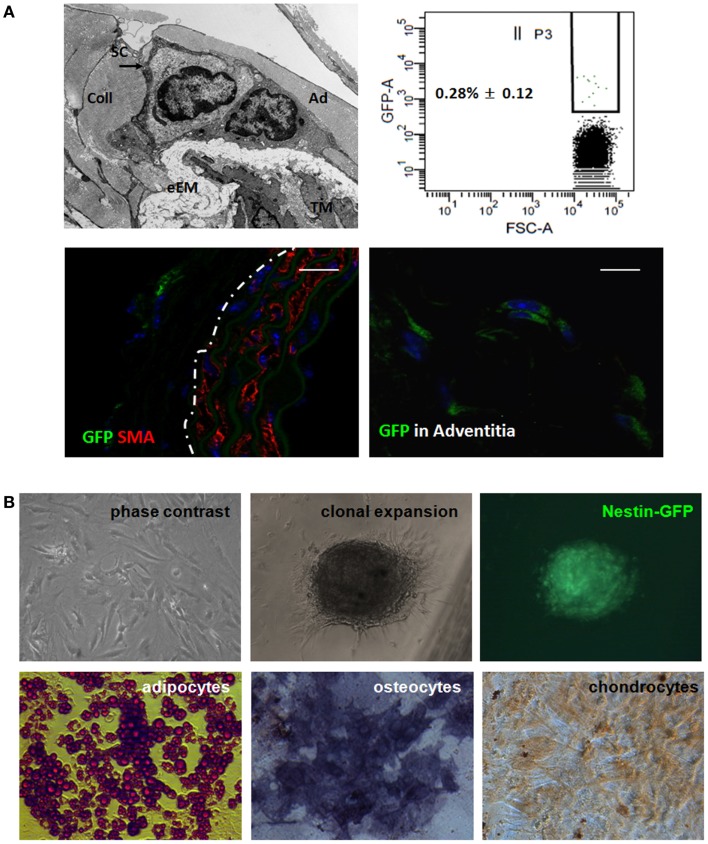
**Nest-GFP(+) cells are localized in the vascular adventitia of murine aorta**. **(A)** Electron microscopic analysis indicates the presence of undifferentiated cells [putative stem cells (SC) in the vasculogenic zone (Ad) of the adventitia **(A)**]. eEM, external elastic membrane; Coll, collagen. *Ex vivo*-isolated aortas from Nest-GFP mice were digested by a short incubation in collagenase solution and sorted by FACS based on the GFP-expression. Immunofluorescence analysis for Nest-GFP(+) MSCs in their native niche was performed using double immunostainings on mouse aorta sections combining antibodies against GFP (green) and SMA (red). Dotted line marks the border between media and adventitia of the aorta wall. Scale bar 20 μm. **(B)** Verification of trilineage differentiation. Clonally expanded Nest-GFP(+) were subjected to *in vitro* differentiation into adipocytes, chondrocytes, and osteocytes. Differentiation was observed within 14 days after induction of differentiation as shown by Oil red staining, by immunostaining for collagen type II (Coll II) and by histochemical staining for alkaline phosphatase (ALP). Magnification 620×.

To gain further insight into the identity of the Nestin(+) cells, we next analyzed whether Nest-GFP(+) cells are also localized in the vascular adventitia of murine aorta under physiological conditions (Figure 6). As expected, in electron microscopic analysis we found undifferentiated cells in the adventitia of mouse aortas (Figure 6A). Immunostaining of murine aorta sections using antibodies against GFP and Nestin identified Nest-GFP(+) cells near by the SMC layer, the tunica media. GFP(+) cells were negative for CD34, a marker for endothelial and hematopoietic progenitors (not shown).

Next, we isolated the GFP(+) cells from the aortic wall by a short collagenase digestion of mechanically minced adventitial tissue fractions prepared from whole *ex vivo*-isolated aortas. Subsequent FACS analysis revealed that 0.28% (±0.12) of the cells in the primary isolate were GFP(+). After a selective adherence on plastic dishes, we further characterized the isolated GFP(+) cells *in vitro* (Figure 6B). *In vitro* clonally expanded cells were Nest-GFP(+) and differentiated into adipocytes, chondrocytes, as well as into osteoblastic cells demonstrating a classical mesenchymal stem cell-like behavior.

**Figure 7:**
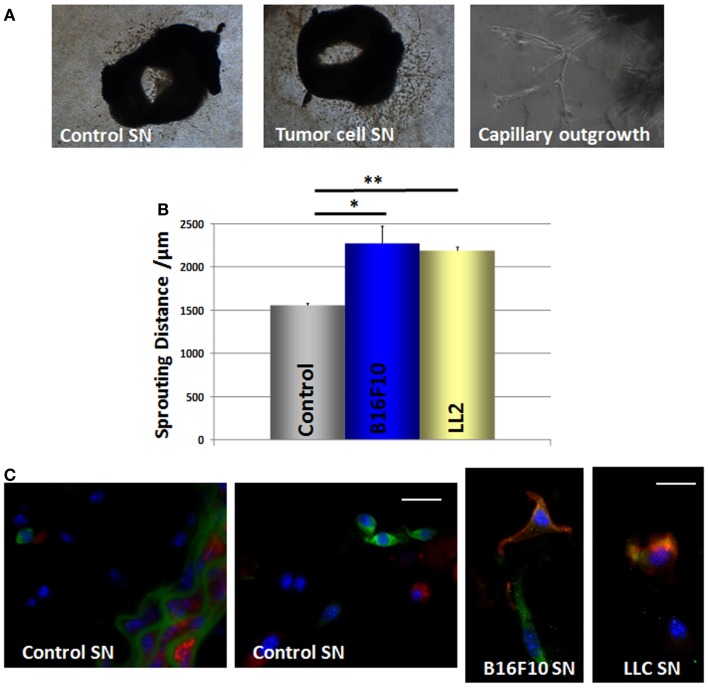
**B16F10 and LLC tumor-cell supernatants increase the mobilization and in-gel sprouting of mouse aorta cells and induce Nest-GFP MPSC differentiation into pericytes**. **(A)** Mouse aorta fragments of Nest-GFP transgenic mice embedded in Collagen-gel were exposed to B16F10 and LLC cell culture supernatants (SN) or control supernatants, respectively. In-gel sprouting and gel invasion was followed by phase contrast microscopy for 14 days. **(B)** The distance of capillary-like sprouts was measured to quantify the invasion. Data are presented as mean ± SD from four independent experiments measured at least two times each. *p ≤ 0.05; **p ≤ 0.005. **(C)** Collagen gels were fixed, paraffin embedded, and tissue sections were subjected for immunofluorescence using antibodies against GFP (green) and SMA (red). Scale bar 10 μm.

We then performed ring assay studies using small mouse aorta fragments to test whether GFP(+) cells can be mobilized from their niche in response to tumor-secreted factors (Figure 7A). Quantitative measurements revealed that cells from the explanted vessels showed a significantly increased in-gel sprouting phenotype whenever aorta fragments were treated with tumor-cell culture supernatants derived from B16F10 and LLC cells (Figure 7B). These findings suggest that tumor-cell-derived soluble factors promote MPSC mobilization and invasion.

GFP(+) cells were found within the Matrigel after 7–14 days of culture indicating that GFP(+) cells are capable to migrate out of the vessel wall whereas the SMC layer seemed to remain intact. Of note, when cultured in the presence of tumor-cell supernatants the GFP(+) cells became also positive for the pericyte/SMC marker SMA whereas untreated GFP(+) cells lacked SMA expression (Figure 7C). This indicates that mobilized GFP(+) cells are capable to differentiate into SMA(+) pericytes and SMCs under the influence of tumor-cell-secreted factors.

## Discussion

Interest is increasing in identifying the origin and the localization of pericytes and SMC in tumor tissue specimens from cancer patients and to gain a better understanding of their role in tumor growth and the outcome of anti-cancer therapies.

Here, we show for the first time that a prominent stabilization of tumor vessels in human colorectal adenocarcinoma metastases under clinical treatment with bevacizumab is achieved by a much more pronounced integration of Nestin(+) cells into the wall of maturing vessels, whereas mature vessels from the tumor-surrounding area or healthy tissue were characterized by decreased Nestin-expression. Moreover we provide evidence that Nestin(+) MPSCs inducing the maturation of tumor vessels do not originate from the BM but presumably reside within the adventitia of adult blood vessels and are mobilized from their niche and differentiated into pericytes and SMC in the presence of tumor-cell-secreted factors.

Our findings suggest Nestin as a novel marker of pericytes and pericyte-like mural cells and thus of vascular maturation. Immunostaining of tissue specimen for Nestin may therefore be of use as a prognostic marker to evaluate the extent of vessel maturation in cancer specimens. Even more important, a selective detection of newly formed tumor vessels within cancer tissues using specific markers raises the possibility of molecular targeted therapy via the inhibition of tumor angiogenesis (21).

In line with our assumption, it was shown that expression of Nestin is specific to developing vascular smooth muscle cells (VSMC) whereas differentiated, postmitotic VSMC are negative for Nestin (22). Generally, expression of the intermediate filament protein Nestin has been detected in repair processes and in various neoplasms and has been associated with immature and angiogenic blood vessels including proliferating vascular endothelial cells (21). In contrast, we did not detect any single Nestin(+) endothelial cell, neither in the murine models nor in different human (tumor) specimen investigated here. One reason for this might be a different staining pattern of the different antibodies used to detect Nestin in different specimen. In addition, no differentiation of hMPSCs to endothelial cells was detected *in vitro* (23, 24).

Consistent with our findings about a role of Nestin(+) tissue-resident MPSCs as major source for pericytes and SMC in vascular remodeling processes, Nestin(+) pericytes have recently been identified as the progenitors of all Leydig cell phenotypes in another Nest-GFP transgenic mouse model. Consistent with our observations, Nestin-expression was also not detected in endothelial cells in that model. These findings implicate that similar to adult stem cells vascular-resident progenitor cells also play a critical role in organ formation (25).

Despite the controversy whether Nestin-expression is restricted to angiogenic endothelial cells or to Nestin(+) stem cells that associate and integrate into newly formed blood vessels, our results still endorse the idea that a Nestin-targeted therapy may be suited to selectively suppress tumor proliferation via inhibition of neovascularization and vessel stabilization in numerous malignancies, including colorectal cancer and melanomas.

Though improved clinical outcomes have been observed when combining anti-angiogenic drugs with chemotherapy for many types of human cancers, clinical efficacy of these treatment strategies has not been as successfully as initially hoped. This may at least be partially due to the fact that anti-angiogenic drugs trigger vascular stabilization involving pericyte-coverage and that pericyte-coverage impairs further tumor vessel regression in response to anti-angiogenic treatment (26). In contrast, the presence of VEGF led to ablation of pericyte-coverage on nascent vascular sprouts and vessel de-stabilization (27). Thus, targeting pericyte recruitment, coverage and function in addition to endothelial cells may be suited to promote progress in anti-angiogenic tumor therapy (28, 29).

Our findings about the origin of vascular-stabilizing pericytes provide a further basis for the design of novel strategies to improve anti-angiogenic therapies. Our data clearly show that (i) Nestin-expression serves as novel selective marker for newly formed tumor blood vessels during the process of vascular remodeling and (ii) that Nest-GFP(+) MPSCs are directly involved in vascular remodeling processes in terms of vascular stabilization, serving as a local source for pericytes and SMC (14).

Up to now, the origin of pericytes in tumors and the molecular mechanisms that govern their recruitment and association with tumor vessels is not well understood. It has been suggested that endothelial-derived PDGF-B triggers the recruitment of pericytes to mature, remodel, and stabilize newly formed vessels so that immature vessels with or without pericytes are formed (30). However, the role of BM-derived vascular progenitor cells vs. tissue-resident stem cells in neovascularization is still controversial. Several reports suggest that adult neovascularization involves BM-derived endothelial progenitor cells (BM-EPCs) providing an alternative source of endothelial cells in the process defined as post-natal vasculogenesis (31, 32). On the basis of these observations BM-EPCs are thought to represent a new and promising target for pro- or anti-angiogenic treatment strategies (33). However, the exact role of these cells in post-natal vasculogenesis and their contribution to vascularization of primary tumors is not yet clear; the respective values range from 50% incorporated BM-EPCs to undetectable numbers (34, 35). In contrast, our findings in tumors grown on reconstituted NestwtBM mice revealed that exclusively SMA(+) pericyte-like cells expressed GFP demonstrating that Nest-GFP(+) pericytes derived from tissue-resident cells and not from the BM stabilize angiogenic vessels in the tumors.

Electron microscopy as well as immunofluorescence analyses on murine aorta sections support the presence of undifferentiated Nest-GFP(+) cells in the adventitia of mouse aortas under physiological conditions. Furthermore, isolated Nest-GFP(+) cells from mouse aortas exhibited typical MSC characteristics. Our *ex vivo* experiments with murine aorta rings showed in addition that these cells can be mobilized from their niche by factors secreted from cultured tumor-cell lines and are capable to differentiate into pericytes and SMC. According to the guidelines, clonally expanded cells adhered on plastic, differentiated into adipocytes, chondrocytes, and osteocytes under certain cell culture conditions (36).

Recently, it has been elegantly shown that Nest-GFP(+) cells in the BM are enriched in mesenchymal stem cell activities and were pericytes-like (37). Further studies are needed to investigate the role of Nestin-expression in developing pericytes as well as during SMC differentiation. There is an urgent need in identifying cells and signaling molecules that are selectively regulated during the process of new vessel formation and vascular remodeling. Here, we suggest that the vascular adventitia serves as a niche for Nest-GFP(+) MPSCs thereby supporting previous studies that the distribution of MSCs throughout the post-natal organism is related to their existence in a perivascular niche (38). It is well known that SMC can arise from multiple sources (39). Lineage tracing studies might help to investigate whether the origin of vascular SMCs is restricted to Nest-GFP(+) MPSCs, which are supposed to reside within the wall of arterial blood vessels as presented here.

In summary, we show here that tissue-resident Nestin(+) MPSCs are more relevant than BM-derived cells for vessel stabilization and serve as a local source for pericytes and SMC. Thus, tissue-resident Nestin(+) MPSCs may be a promising target for anti-angiogenic therapies. The identification of proteins mediating the recruitment of local Nestin(+) cells with potential stem cell character to angiogenic blood vessels and of their differentiation into mural cells may allow the definition of new therapeutic strategies to counteract pathologic vascular remodeling and to reduce tumor resistance against anti-angiogenic drugs.

## Conflict of Interest Statement

The authors declare that the research was conducted in the absence of any commercial or financial relationships that could be construed as a potential conflict of interest.
